# Elevated fatty acid β-oxidation by leptin contributes to the proinflammatory characteristics of fibroblast-like synoviocytes from RA patients via LKB1-AMPK pathway

**DOI:** 10.1038/s41419-023-05641-2

**Published:** 2023-02-09

**Authors:** Jing Wei, Xinxin Huang, Xing Zhang, Guanghong Chen, Cheng Zhang, Xinyang Zhou, Jingjing Qi, Yan Zhang, Xia Li

**Affiliations:** 1 Basic Medical Sciences, Dalian Medical University, Dalian, China; 2 Department of Rheumatology, The Second Hospital Dalian Medical University, Dalian, China

**Keywords:** Rheumatoid arthritis, Rheumatoid arthritis

## Abstract

Fibroblast-like synoviocytes (FLS) maintain chronic inflammation leading to joint destruction in rheumatoid arthritis (RA). Fatty acid β-oxidation (FAO) regulates cell function. Here, we aimed to investigate the effect of FAO enhanced by leptin on the characteristics of RA-FLS and elucidate the potential metabolic mechanism. Key enzymes involved in lipid metabolism were detected with qPCR in HSF, MH7A cell line and isolated RA-FLS treated with RA or healthy control (HC) serum. In some experiments, FAO inhibitor, etomoxir (ETO) or anti-leptin antibody were added into serum-treated RA-FLS. In other experiments, RA-FLS were stimulated with leptin together with ETO or AMP-activated protein kinase (AMPK) inhibitor compound C (CC) or silencing liver kinase B1 (LKB1). Cell proliferation, proinflammatory factor production, pro-angiogenesis, chemoattractive potential, FAO-related key enzymes, AMPK and LKB1 in FLS were analyzed. FAO-related key enzymes were evaluated in serum-treated RA-FLS with or without anti-leptin antibody. Related functions of leptin-stimulated RA-FLS were examined in the presence or absence of ETO. AMP-activated protein kinase (AMPK) and liver kinase B1 (LKB1) in leptin-stimulated RA-FLS were tested with western blot. Activation of AMPK in leptin-stimulated RA-FLS was detected after silencing LKB1. We found that MH7A cell line and RA serum-treated FLS exhibited upregulated FAO, and ETO could inhibit the proinflammatory phenotypes of RA-FLS. The addition of anti-leptin antibody suppressed the elevation of FAO mediated by RA serum. More importantly, leptin promoted the proinflammatory characteristics of RA-FLS, which was reversed by ETO. Leptin activated AMPK by upregulating LKB1. CC impaired leptin-induced CPT-1A expression in RA-FLS. Our study uncovers that elevated FAO mediated by leptin drives abnormal function of RA-FLS and suggests leptin or FAO inhibition may serve as a promising therapeutic strategy for RA.

## Introduction

Rheumatoid arthritis (RA) is an autoimmune disorder characterized by synovial hyperplasia, pannus formation, bone and cartilage destruction. Agents with anti-inflammatory and anti-bone destruction activity may have therapeutic potential for RA [1]. Recent advance in understanding the biology of fibroblast-like synoviocytes (FLS) provides novel insights into RA mechanisms [2]. FLS represent the primary stromal cells and play an important role in the maintenance of synovial homeostasis [3, 4]. FLS exhibit an aggressive phenotype, predisposing them to be involved in an inflammatory positive feedback loop in response to the elements from the synovial environment in RA pathologenesis [5, 6]. Targetting FLS can potentially complement the current understanding of RA etiology. High levels of cytokines, growth factors and infiltrating inflammatory cells, as well as hypoxia conditions in RA joints contribute a lot to the formation of pro-inflammatory environment and play key roles in FLS activation and aggressive phenotypes [7, 8]. It is imperative to understand the molecular pathways sustaining FLS aggressive phenotype.

Fatty acid β-oxidation (FAO) is gradually proved to be involved in the regulation of immune-mediated diseases. As reported in the literature, RA synovial milieu severely alters fatty acid metabolism and upon infiltration into the RA synovial tissue, monocytes alter their fatty acid metabolic that in turn can support CCL20-mediated inflammation in RA [9]. Abnormal fatty acid oxidation is an important part for the maintenance of cell function, since related researches mainly focus on immune cell fate determination and cancer cell fate determination [10, 11]. However, whether FLS attain a unique phenotype through catabolic metabolism such as FAO remains to be explored. There has been a great interest in probing the regulatory effect of FAO on RA-FLS.

The critical role of RA-FLS in synovial lesions and, more recently, in metabolic regulation has been recognized [12]. Leptin, increased in serum and synovial fluid of RA patient, is the main adipokine which exerts potent modulatory actions in the pathophysiology of RA [13, 14]. The instrumental role of leptin as a paracrine factor on cell function and differentiation has been demonstrated [15]. Our previous results showed that leptin induced RA-FLS migration and angiogenesis by increased reactive oxygen species (ROS) production [16]. The evident role of leptin is to regulate energy homeostasis and food intake. However, it also has pleiotropic functions [17]. Therefore there is a strong interest in whether leptin is involved in the regulation of FAO to sustain RA-FLS functional variations [18].

Therefore, in the present study, we screened the differentially expressed genes (DEG) from two gene expression profiles and analyzed DEG involved in glycolipid metabolism. Our team takes this as the starting point and tries to investigate the role of leptin in RA-FLS from FAO metabolic pathway and elucidate the underlying mechanism as well as therapy target.

## Results

### FAO-related genes were elevated in RA-FLS

**Fig. 1:**
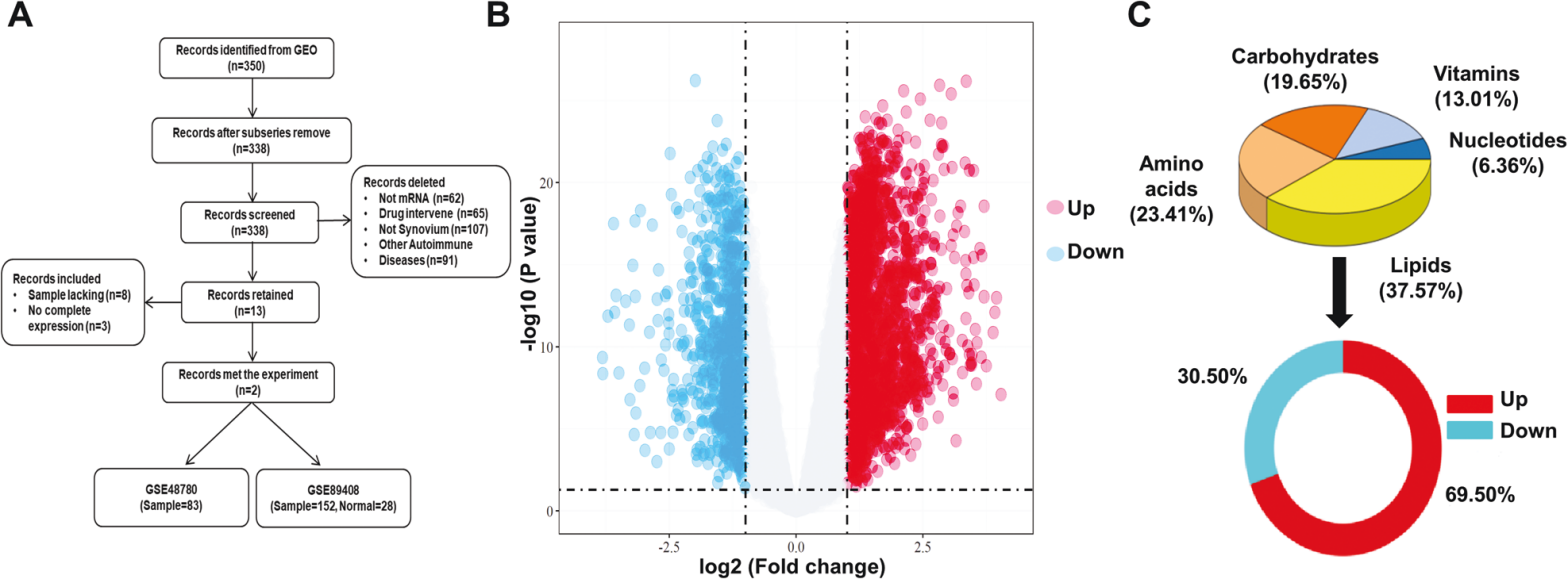
Analysis of differentially expressed genes (DEGs) related to major metabolism in synovial tissues from RA and normal control. **A** The flow chart of obtaining and screening datasets from the GEO. **B** DEGs of synovial tissues between RA patient and normal control were visualized with volcano plot by ggplot2. Upregulated genes were shown in red and down-regulated were shown in blue. **C** Intersection analysis results of DEGs and metabolic genes. According to the corresponding metabolites, the metabolic process consisted of five parts, which are expressed in percentage.

Differentially expressed genes (DEGs) of synovial tissue between RA patients and healthy controls (HC) were determined from two publicly available datasets. There were 2453 upregulated genes and 849 down-regulated genes in a total of 3302 DEGs (Fig. 1A, B). We screened metabolism-related genes from DEGs and found lipid metabolism-related genes accounted for the largest proportion (37.57%), and the majority of them (69.5%) were upregulated (Fig. 1C). The preliminary analysis suggest that abnormal fatty acid metabolism might have a hand in RA pathological process.

**Fig. 2:**
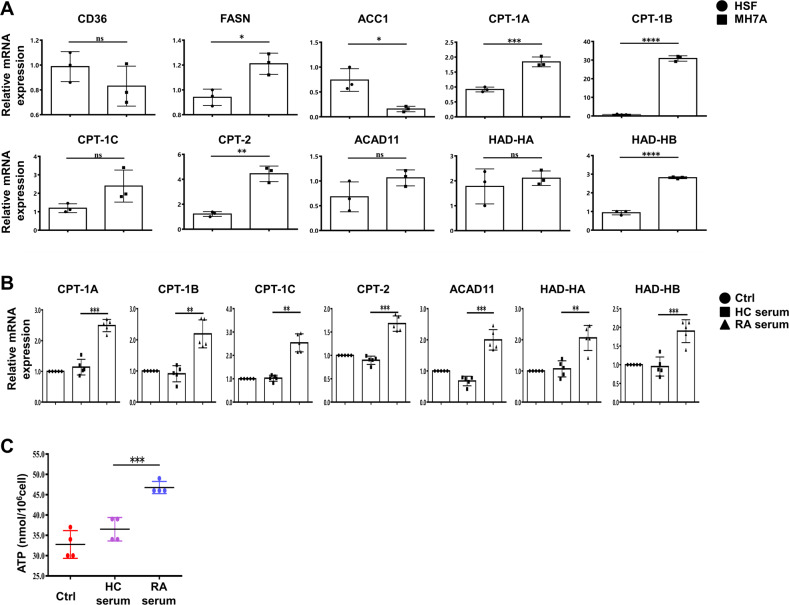
FAO-related genes were elevated in RA-FLS. **A** Total RNA was isolated and the mRNA expression of the indicated genes was quantified by real-time PCR relative to housekeeping mRNA expression in HSF and MH7A (*n* = 3). **B** Expression of FAO-related genes in RA-FLS treated with vehicle (5% FBS stimulation group, Ctrl) or 5% serum from RA patients or healthy controls (HC) for 24 h (*n* = 5). **C** Intracellular ATP contents in RA-FLS stimulated by vehicle, HC and RA serum for 24 h (*n* = 5). Data are means ± SEM of three independent experiments; *P < 0.05, **P < 0.01, ***P < 0.001, ****P < 0.0001, ns means no significance versus the control group. Abbreviations: FASN Fatty acid synthase, ACC1 Acetyl-CoA carboxylase-1, CPT-1 Carnitine palmitoyltransferase 1, CPT-2 Carnitine palmitoyltransferase 2, ACAD11 Acyl-CoA dehydrogenase 11, HAD-HA Hydroxyacyl-CoA dehydrogenase HA, HAD-HB Hydroxyacyl-CoA dehydrogenase HB.

To confirm above results from database analysis, giving thought to FLS is not only the main resident cell of synovial tissue but also key proinflammatory actor, we made a comparison of major lipid metabolism-related enzymes between human rheumatoid FLS cell line (MH7A) and skin fibroblasts line (HSF). mRNA expression of lipid metabolism-related enzymes including fatty acid uptake (CD36), fatty acid synthesis (FASN, ACC1) and FAO (CPT-1A/1B/1 C, CPT-2, ACAD11, HAD-HA, HAD-HB) were significantly higher in MH7A compared with HSF (Fig. 2A). Then we isolated primary FLS from synovial tissues of RA patients. After identification (Fig. S1), FLS were treated with vehicle, serum from HC or RA patient respectively. Differential expression of FAO related enzymes and intracellular ATP content were identified. The results indicated that expression of FAO related enzymes including CPT-1A/B/C, CPT-2, ACAD11, HAD-HA/HB and intracellular ATP content were elevated in RA serum-stimulated group compared to control and HC serum-treated group (Fig. 2B, C). Together, these data indicate that FAO metabolic pathway was enhanced in RA-FLS.

### Elevated FAO contributed to the inflammatory phenotype of RA-FLS

**Fig. 3:**
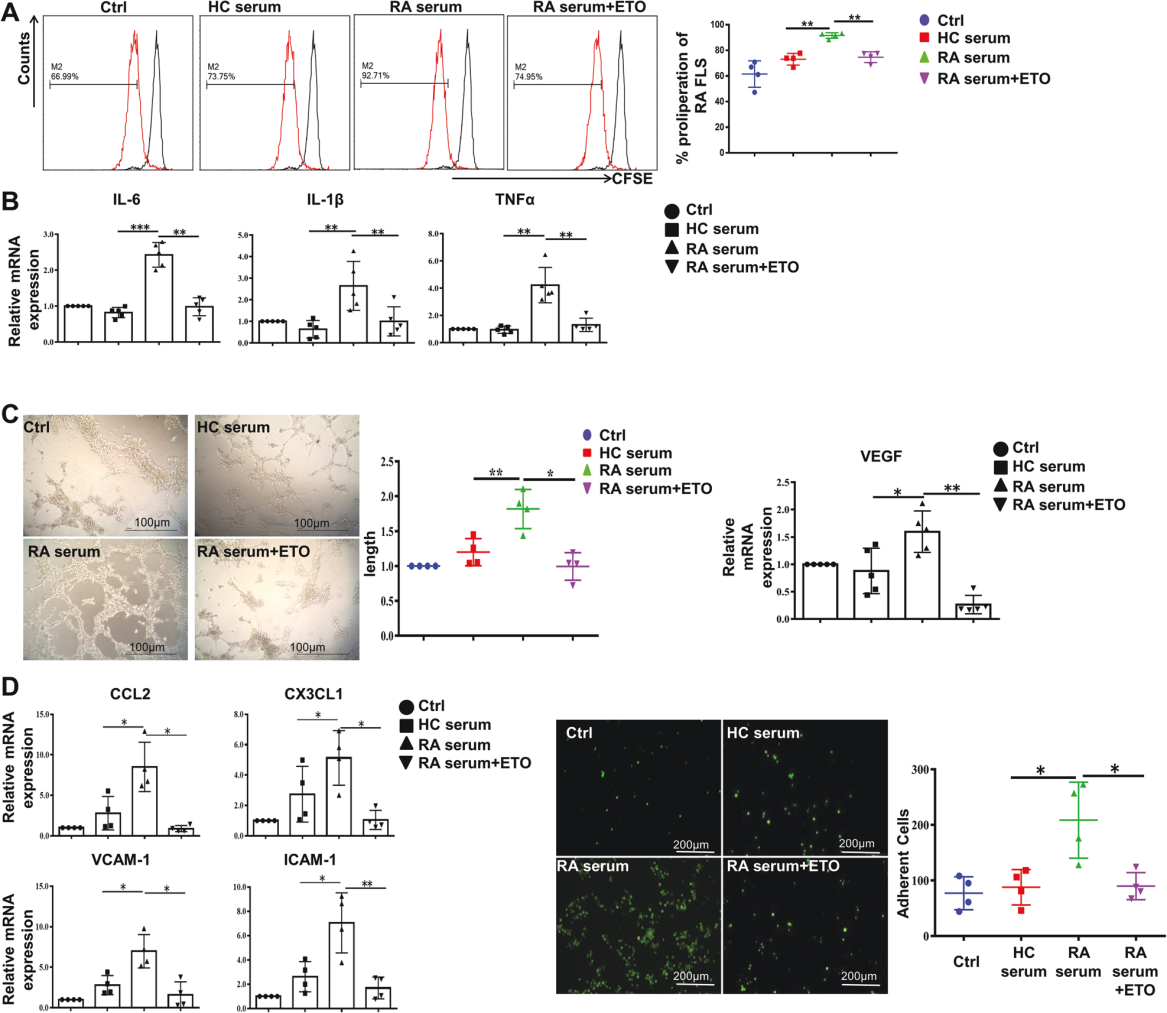
Elevated FAO contributed to the inflammatory phenotype of RA-FLS. RA-FLS were treated with vehicle, serum from HC, and serum from RA patient with or without ETO (100 μM) for 24 h. **A** CFSE-based assay was employed to detect the proliferation of RA-FLS with FACS analysis (*n* = 4). **B** The relative mRNA expression of IL-6, IL-1β and TNF-α was determined by real-time PCR analysis (*n* = 5). **C** Representative images of capillary-like structures were captured and quantitative analysis of total tube length was checked (left, scale bar = 100 μm, *n* = 4). Gene expression of VEGF in RA-FLS was detected (right, *n* = 5). **D** Gene expression of CCL2, CX3CL1, VCAM-1 and ICAM-1 in RA-FLS exposed to indicated stimulations was determined (left). Images were captured with a fluorescence microscope. Monocytes adhered to RA-FLS were described in green (right, scale bar = 200 μm, *n* = 4). Data are means ± SEM of three independent experiments, statistical significance was determined as **P < 0.01 and *P < 0.05 compared with control.

Carnitine palmitoyl transferase 1 (CPT1) constitutes a rate-limiting step of FAO by shuttling long-chain fatty acids into mitochondria [19], in order to investigate the role of FAO in RA-FLS, we treated FLS with RA serum in the presence or absence of irreversible CPT1 inhibitor etomoxir (ETO) and detected the changes of proinflammatory phenotypes in RA-FLS. The results showed that proliferation of RA-FLS (Fig. 3A), mRNA expression of pro-inflammatory cytokines (IL-6, IL-1β, TNF-α) (Fig. 3B) were obviously decreased, together with lower expression of VEGF and more declined pro-angiogenesis role of HUVEC (Fig. 3C) following FAO inhibition with ETO.

RA-FLS possess the ability of recruiting monocytes from circulation into RA synovium by secreting chemokines and adhesion molecules, and subsequently aggravates inflammation of involved joints. Further we analyzed adhesion and chemotaxis related-genes and found that VCAM-1, ICAM-1, CCL2 and CX3CL1 were upregulated in RA-FLS stimulated with serum from RA compared to healthy control, which could be reversed by adding ETO to RA serum. In addition, immunofluorescence test confirmed that ETO decreased RA serum-induced monocyte-adhesion to FLS (Fig. 3D). These results indicate that elevated FAO in RA-FLS may contribute to their abnormal activities in RA inflammatory microenvironment.

### Leptin was critical for FAO pathway in RA-FLS

Adipokines possess major metabolic and endocrine functions implicated in systemic energy, glucose, and lipid homeostasis. Our previous study has verified that leptin, one of the classical adipokines, was increased and positively correlated to RA disease activity [20]. In this study, we wondered whether leptin was involved in the regulation of FAO in RA-FLS?

**Fig. 4:**
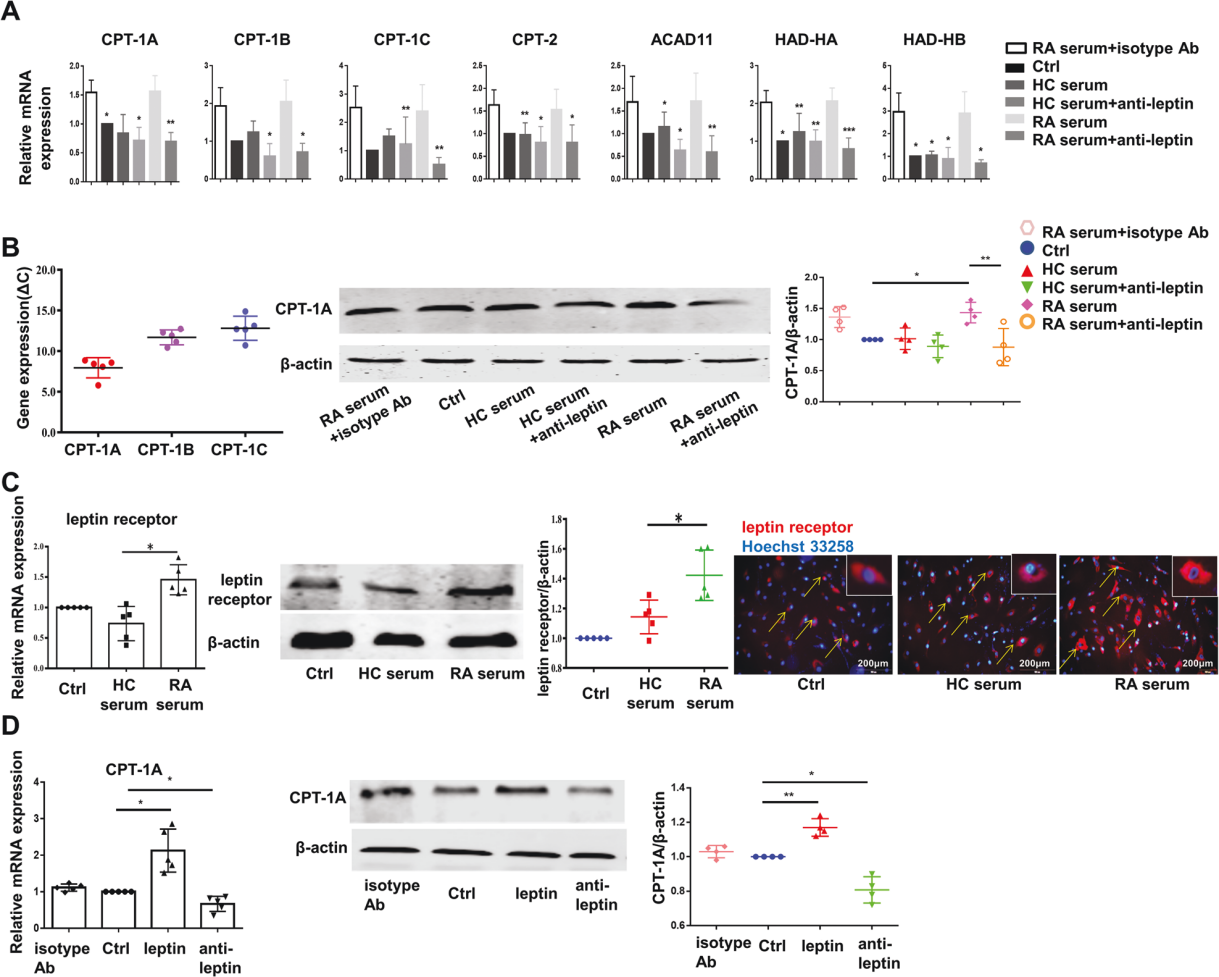
Leptin was critical for FAO pathway in RA-FLS. **A** RA-FLS were stimulated with vehicle, serum from RA patient and HC with or without anti-leptin antibody (500 ng/mL), together with serum from RA patient and isotype antibody (500 ng/mL). Cells were harvested at 24 h and mRNA expression of the indicated genes was determined with real-time PCR analysis (*n* = 5). **B** Measurement of CPT-1A, CPT-1B and CPT-1C mRNA expressions in RA-FLS by real-time PCR (left, *n* = 5). RA-FLS were treated with vehicle, serum from HC with or without leptin, serum from rheumatoid arthritis with or without leptin antibody, isotype antibody, and levels of CPT-1A were determined by western blot (*n* = 4). **C** Expression of leptin receptor was assessed in RA-FLS treated by vehicle, serum from HC and RA patient with real-time PCR analysis (*n* = 5), western blot and immunofluorescence assay (scale bar = 200 μm, *n* = 4). **D** CPT-1A was tested with real-time PCR in RA-FLS stimulated by leptin (100 ng/mL), anti-leptin antibody (500 ng/mL) and isotype antibody (500 ng/mL) for 24 h, respectively (*n* = 5). CPT-1A protein expression was also detected at 48 h after indicated stimulations (*n* = 4). Data are means ± SEM of three independent experiments, statistical significance was determined as **P < 0.01, *P < 0.05, ns means no significance compared with control.

RA-FLS were treated with serum from RA patient or HC in the presence or absence of leptin neutralizing antibody which limits the role of leptin. The result indicated that human leptin neutralizing antibody (anti-leptin) could reverse the rising expression of FAO-related enzymes elicited by RA serum, including CPT-1A/B/C, CPT-2, ACAD11 and HAD-HA/HB (Fig. 4A). CPT-1 consists of three subtypes [21]. CPT-1A, a subtype of the CPT1 transport system [22], was found to be the most predominant in RA-FLS (Fig. 4B, left). In addition, we also analyzed the protein level of CPT-1A in RA-FLS treated with RA serum or healthy control serum in the presence or absence of leptin neutralizing antibody. In addition, CPT-1A protein expression with western-blot analysis of FLS was increased in RA serum-stimulated group, which could be inhibited after the addition of leptin neutralizing antibody(Fig. 4B, right).

Since leptin exerts its biological action through binding to leptin receptor, we tested mRNA and protein expression of leptin receptor in RA-FLS stimulated by RA or healthy control serum with real-time PCR, western blot and immunofluorescence staining. The results indicated that both gene and protein expression of leptin receptor were elevated in RA-FLS stimulated by RA serum in relation to HC serum (Fig. 4C). Furthermore, as shown in Fig. 4D, anti-leptin antibody reversed the increased CPT-1A mRNA and protein levels induced by leptin. Taken together, these results indicated that leptin might be a critical factor leading to enhancement of FAO in RA-FLS.

### Leptin-upregulated FAO was responsible for inflammatory phenotypes of RA-FLS

**Fig. 5:**
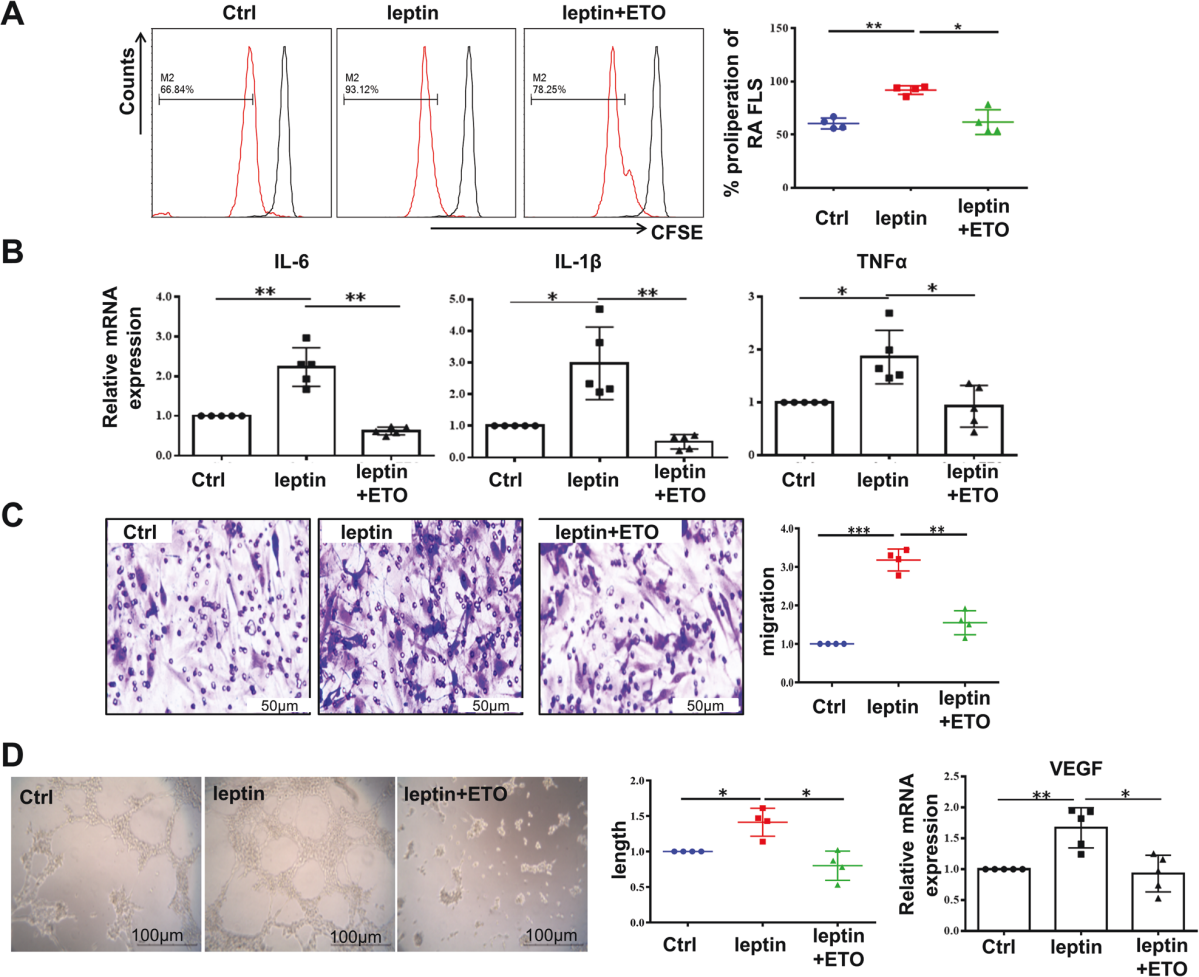
Leptin-upregulated FAO was responsible for inflammatory phenotypes of RA-FLS. RA-FLS were handled by vehicle, leptin (100 ng/mL) with or without ETO (100 μM) for 24 h. RA-FLS were labeled with CFSE and analyzed by flow cytometry for proliferation (**A**, *n* = 4). **B** Expression of IL-6, IL-1β and TNF-α in RA-FLS was tested with real-time PCR analysis (*n* = 5). **C** Transwell migration assay was carried out to evaluate migration potential of RA-FLS (scale bar = 50 μm, *n* = 4). **D** Supernatant derived from RA-FLS with indicated treatment was added into the culture system of HUVEC. Representative images of capillary-like structures and quantitative analysis of the total tube length were examined at 6 h (scale bar = 100 μm, *n* = 4). Expression of VEGF was evaluated by real-time PCR at 24 h. Data are means ± SEM of three independent experiments, statistical significance was determined as ***P < 0.001, **P < 0.01, *P < 0.05 compared with control.

To further validate the role of upregulated FAO pathway induced by leptin in RA-FLS, we treated RA-FLS by leptin with or without ETO. The result illustrated that the proliferative capacity, together with the release of pro-inflammatory cytokines (IL-6, IL-1β and TNF-α) which formed a local inflammatory malignant cycle of synovium, were enhanced in leptin-irritated RA-FLS. However, administration with ETO reversed the above-mentioned functions (Fig. 5A, B). In addition, abnormal migration ability of FLS is another important property closely related to RA synovial lesions. Transwell migration assay proved that cell motility was increased upon leptin-stimulation, which could also be inhibited by supplement with ETO (Fig. 5C). Furthermore, ETO suppressed vascular structure formation of HUVEC and VEGF expression which were enhanced by leptin (Fig. 5D). Based on the aforementioned results, we drew a conclusion that FAO elevation by leptin in RA disease microenvironment conduced a lot to the pro-inflammatory activities of FLS.

### Leptin-upregulated FAO favored FLS interactions with other effector cells of RA

**Fig. 6:**
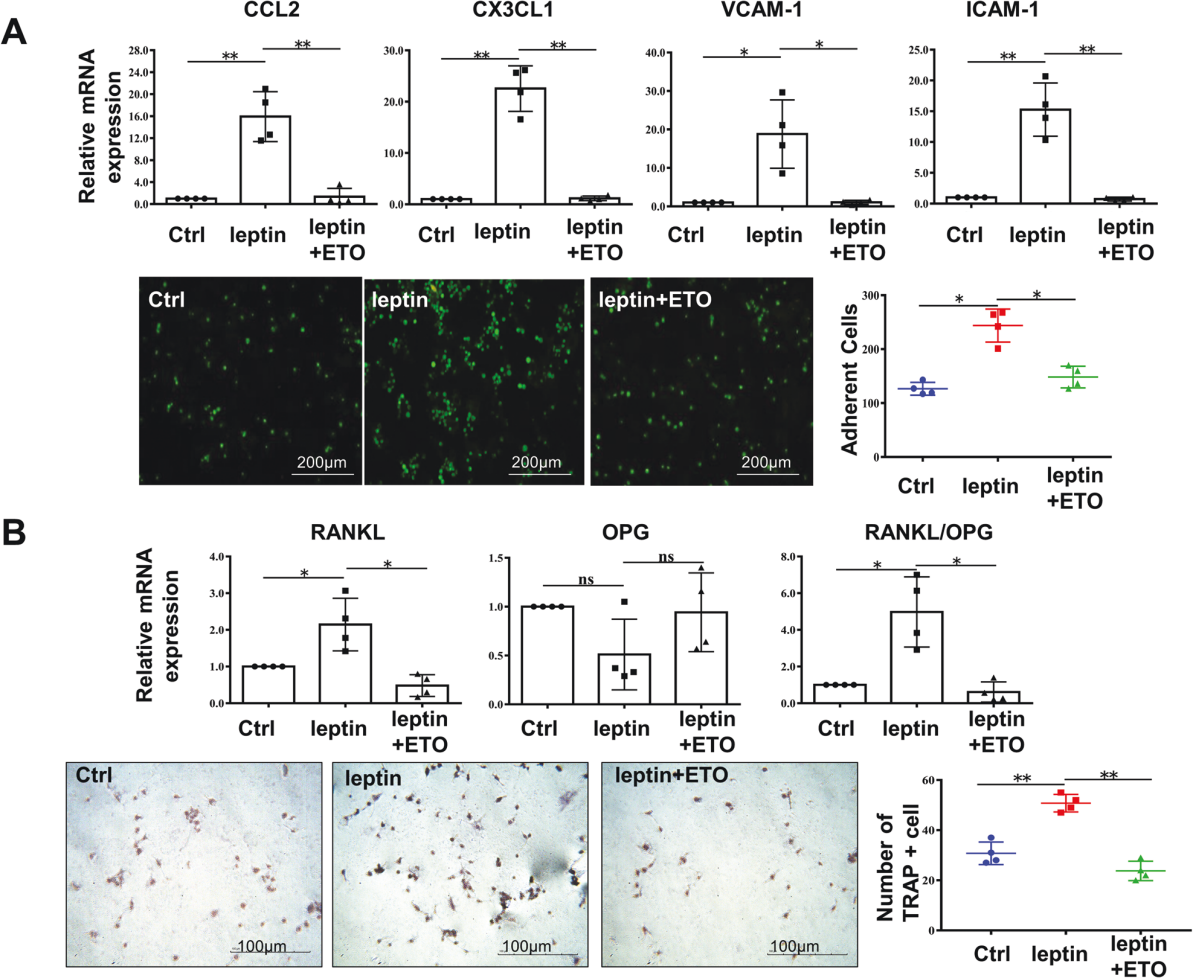
Leptin-upregulated FAO favored FLS interactions with other effector cells of RA. RA-FLS was stimulated with leptin in the prsence or absence of ETO for 24 h. **A** Real-time PCR was performed to compare the expression of CCL2, CX3CL1, VCAM-1, ICAM-1 (*n* = 4). Comparison of THP-1 monocyte adhesion to RA-FLS was detected and images were captured with a fluorescence microscope (scale bar = 200 μm, *n* = 4). **B** RANKL, OPG expression and RANKL/OPG ration were assessed with real–time PCR analysis (*n* = 4). TRAP-positive multinucleate cells were identified and counted after THP-1 monocyte was stimulated with supernatant from RA-FLS exposed to corresponding treatment. TRAP-positive cells were captured under a light microscope (scale bar = 100 μm, *n* = 4). Data are means ± SEM of three independent experiments, statistical significance was determined as **P < 0.01, *P < 0.05, ns means no significance compared with control.

In addition to its direct role in RA pathogenesis, FLS also interacts with other neighboring cells through paracrine networks, including monocyte recruitment to synovium mainly by production of chemotactic factors in the propagation of rheumatoid process [4]. Peripheral monocytes were thought to be the major source of osteoclasts under inflammatory environment and the crucial role of osteoclasts in arthritic bone degradation has also been demonstrated [23]. A large number of hyper differentiated osteoclasts were accumulated at the bone injury site of RA, particularly at the bone where the proliferating synovium invaded. RANKL has been identified as the main inducing factor responsible for osteoclast differentiation [24]. In leptin and ETO co-treatment group, the expression of CCL-2, CX3CL1, VCAM-1, ICAM-1, RANKL, together with RANKL/OPG ratio were descended in contrast to leptin-stimulated group. Moreover, ETO treatment reversed the adhesion potential of monocytes to RA-FLS which was enhanced by leptin (Fig. 6A, B). We concluded from these results that FAO of RA-FLS powered by leptin contributed a great deal to monocyte recruitment and osteoclast-like cell differentiation.

### LKB1-AMPK signal pathway was crucial for leptin-elevated FAO in RA-FLS

**Fig. 7:**
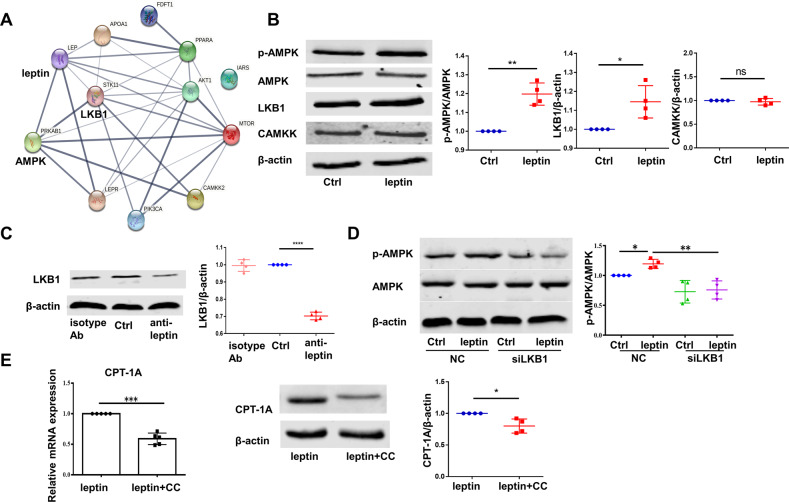
LKB1-AMPK signal pathway was the crucial for leptin-induced FAO in RA-FLS. **A** Protein–protein interactions (PPI) among leptin, leptin receptor and major metabolic pathways was consturcted. Only the PPI with the confidence of >0.4 was kept. **B** AMPK total and phosphorylated protein at T172 (pAMPK), LKB1 and CAMKK protein levels were compared in RA-FLS treated in the presence or absence of leptin by western blot (*n* = 4). **C** Assessment of LKB1 protein level in RA-FLS processed with vehicle, anti-leptin antibody and isotype antibody by western blot (*n* = 4). **D** AMPK total and pAMPK in NC or down-regulation of LKB1 with siRNA in the presence or absence of leptin was detected at 48 h (*n* = 4). **E** CPT-1A gene (*n* = 5) and protein (*n* = 4) levels in RA-FLS stimulated by leptin with or without compound C (CC, 5 μM) was evaluated. Data are means ± SEM of three independent experiments, statistical significance was determined as **P < 0.01, *P < 0.05, ***P < 0.001, ****P < 0.0001, ns means no significance compared with control.

We will further explore the potential pathway-molecules involved in leptin-induced FAO. We first assessed the potential interactions among leptin, leptin receptor and major metabolic pathway molecules, including mammalian target of rapamycin (mTOR), phosphoinositide 3-kinase (PI3K), AKT (known as protein kinase B or PKB), AMP-activated protein kinase (AMPK), insulin receptor substrate (IRS), peroxisome proliferator-activated receptors (PPAR), apolipoprotein A1 (APOA1), farnesyl-diphosphate farnesyl transferase 1 (FDFT1), liver kinase B1 (LKB1) and calmodulin-dependent protein kinase kinase β (CAMKKβ) through protein–protein interactions (PPIs) network. As indicated in Fig. 7A and Table S3, leptin and its receptor were linked most strongly with AMPK pathway.

On the basis of above analysis results, we examined phosphorylation of AMPK (p-AMPK) in RA-FLS treated with or without leptin and found that leptin promoted the activation of AMPK (Fig. 7B). LKB1 was identified as the critical upstream kinase required for AMPK activation [25]. CaMKKβ also possessed the AMPK-activated potential in certain cell types [26]. Afterwards, we investigated whether LKB1 and/or CaMKKβ could function as upstream-activator of AMPK in RA-FLS. Protein level of LKB1 was increased significantly than CaMKKβ, which was basically unchanged after leptin stimulation, and the addition of leptin neutralizing antibody could reverse the elevated LKB1 level (Fig. 7B, C). What’s more, silencing LKB1 with si-1261 (The interfering effect was confirmed by RT-PCR and western blot, as shown in Fig. S2) decreased leptin-induced AMPK phosphorylation (Fig. 7D). We also verified the relationship between AMPK and FAO. After compound C (CC), a widely used as AMPK inhibitor, was added into leptin-treated RA-FLS, both the gene and protein levels of CPT-1A were obviously down-regualted (Fig. 7E). Taken together, we draw the preliminary conclusion that leptin-induced FAO in RA-FLS was dependent on LKB1-AMPK pathway.

## Discussion

**Fig. 8:**
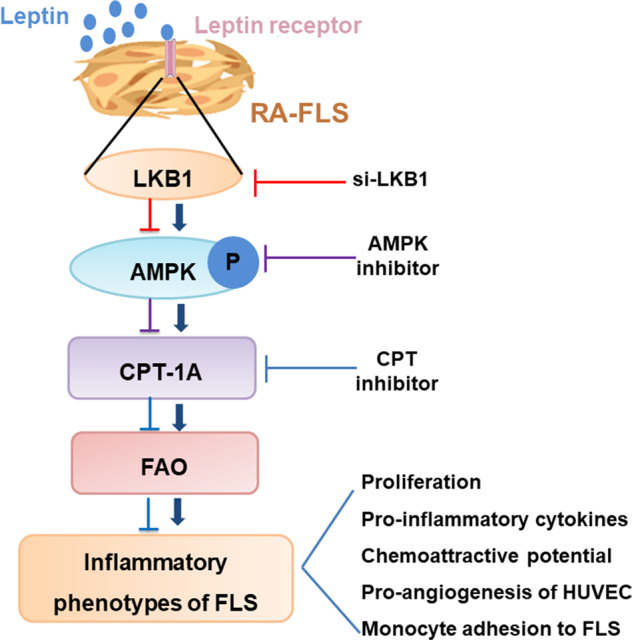
A proposed model of how leptin activates FAO in RA-FLS through LKB1-AMPK pathway. FLS play an important role in the pathogenesis of RA. Our results show that leptin-LKB1/AMPK pathway promots the maintenance of pro-inflammatory characteristics in FLS through upregulating cellular fatty acid β-oxidation (FAO). Blocking FAO and/or depleting leptin will contribute to the alleviation of synovial inflammation elicited by FLS.

Our study identifies a novel leptin-induced, LKB1/AMPK/CPT-1A-dependent FAO pathway as critical roles for FLS dysfunction involved in RA pathogenesis (Fig. 8). We present evidence that FAO-related enzymes were highly expressed in FLS stimulated with RA serum and enhanced FAO was responsible for RA abnormal functions. More importantly, we found that leptin is the main factor contributing to the upregulation of FAO in proinflammatory environment of RA. Further, we verified that this upregulated FLS-FAO was mediated by leptin through the activation of LKB1-AMPK pathway. The results suggest that leptin and its upregulated FAO may be a novel markers of disease and possess a potential therapeutic target in inflammation-related functions of RA-FLS.

RA-FLS invasive characteristics are related to a series of pathological processes, such as overproliferation, release of proinflammatory mediators, interactions with immune and non-immune cells, direct or indirect joint destruction [27–29]. It is demonstrated that lipid metabolism can promote breast cancer stemness and chemoresistance [19]. In this study, we are committed to clarify the regulatory roles of intracellular metabolism on RA-FLS abnormalities from a relatively new perspective. We found that abnormal fatty acid metabolism might have a hand in RA pathological process during the DEGs analysis. We confirmed that FAO-related enzymes were increased in RA serum-stimulated FLS.

Recent researches demonstrate that the RA synovial environment has profound and distinct effects on the metabolic profile of human monocytes, particularly in fatty acid metabolism and FAO has been verified to regulate the polarization of murine macrophage [8, 30]. While other studies proposed that FAO has a non-essential role in M2 polarization in human macrophages [31], leaving us to question what roles FAO may have on non-immune cells. Here we confirmed that enhanced FAO could bring about abberant chacteristics of RA-FLS, including the proliferation, exacerbated release of pro-inflammatory mediators, facilitated HUVEC capillary-like structure formation, together with the recruitment of monocytes, which were closely related to RA. This was further verificated that ETO treatment to inhibit FAO had been shown to reverse abnormal functions RA-FLS. Our data imply that FLS may require fatty acid catabolism to promote its dysfunction in RA condition.

It is indispensable to explore the key factor leading to enhanced fatty acid oxidation in RA milieu. Leptin has been considered a link between the neuroendocrine and immune systems [32, 33], which exhibits proinflammatory and pro-catabolic actions on cartilage [34–36]. However, Wang et al. showed that leptin-JAK/STAT3 promoted breast cancer stemness and chemoresistance via upregulating lipid metabolism through FAO [19], providing new light to better understanding of the role of lipid metabolic reprogramming. Our and other’s studies have found that leptin is elevated in RA serum and synovial fluid and correlated to disease activity [37]. Our previous researches still showed that leptin could elevate RA peripheral CD4^+^CXCR5^+^ ICOS^+^ Tfh production via IL-6 secretion through STAT1 and STAT3 pathways [20]. We also put forward that leptin promoted RA-FLS migration by ROS overproduction and enhanced HUVECs tube formation in a ROS/HIF-1α-dependent manner [16]. Here, we confirmed that leptin upregulated FAO of RA-FLS via increased CPT-1, which contributes to the FLS dysfunction.

AMPK is a highly conserved kinase that exists widely in eukaryotic cells. AMPK can be activated under stimulation of adverse factors including nutritional deficiency, metabolic disorder, hypoxic ischemia, and oxidative stress [38]. The activation of AMPK is directly regulated by upstream proteins, the main upstream proteins of AMPK are LKB1 and CaMKK [39]. Our data illustrate that leptin exerts modulatory effect on FAO mainly by binding to its receptor through activating LKB1/AMPK pathways. Our results favor the opinion that targeting FAO/leptin can effectively inhibit abnormal function of FLS in RA pathogenesis.

## Materials and methods

### Data collection

GEO, a public National Center for Biotechnology Information (NCBI) database, was used to obtain the mRNA expression datasets in synovial tissue of RA patients and normal controls (GSE48780, GSE89408). The datasets of observing mRNA expression were described in supplementary Table S1. All of the datasets were downloaded in raw data file format.

### Identification of differentially expressed genes

Background correction, standardization and expression value calculation were performed on the original dataset from GEO using the R package. |log2(FC) | ≥ 1 and adjust *p*-values < 0.05 were defined as the screening criteria for DEGs. Volcano maps of DEGs were constructed using the R package “ggplot2”.

### Screening of metabolic differential genes

Metabolic genes set data was collected from MSigDB (http://www.broadinstitute.Org/gsea/msigdb) [40]. We compared the RA synovial tissue differential genes and metabolic genes derived from the above operations, and used MSigDB to find the crossing over genes.

### Human tissue samples, serum and cell lines

Synovial tissue specimens used for culturing RA-FLS were obtained from patients (*n* = 5; age, mean/range: 50/21–65 years; sex, no. female/male: 4/1; duration, mean/range: 25/10–50 years; ACPA, no. positive/no. negative: 5/0; DAS28, mean/range: 5.41/3.53-8.51.) during total knee replacement surgery or arthroscopy. All patients involved in this study fulfilled the American College of Rheumatology 1987 criteria for RA [41]. And serum from RA patients (*n* = 5) and healthy controls (*n* = 5) were collected from the Clinical Laboratory of the Second Affiliated Hospital of Dalian Medical University.

Besides human skin fibroblasts (HSF), Human Umbilical Vein Endothelial Cells (HUVEC), THP-1 and MH7A were purchased from Fein Biological Co., Ltd., Wuhan, China. All cell lines were authenticated by DNA (STR) profiling and tested for mycoplasma contamination before the start of this study. The study protocols and consent forms were approved by the Institutional Medical Ethics Review Board of Dalian Medical University (IRB#2018-061). All patients gave written informed consent.

### Cell culture and treatment

RA-FLS, HUVEC, HSF and MH7A were cultured in DMEM supplemented with 10% fetal bovine serum (FBS) (Thermo Fisher Scientifific, Waltham, MA, USA). THP-1 were cultured in RPMI1640 (Gibco) supplemented with 10% FBS. Cell lines were used from three to five passages in this experiment. All cells were cultured in medium supplemented with penicillin (50 U/mL) and streptomycin (50 μg/mL) and maintained in an incubator with 5% CO_2_ at 37 °C.

FLS were cultured in DMEM medium supplemented with 5% (v/v) FBS, 5% healthy control serum and 5% RA serum (To avoid individual differences, the sera from 5 healthy controls or RA patients were combined into a sample.) after overnight serum starvation. Etomoxir (ETO, Sigma Adrich) was added into serum-treated RA-FLS. In order to block the effect of leptin, RA-FLS were handled by serum from HC or RA patients with or without human leptin antibody (anti-leptin, R&D Systems) and mouse IgG1 isotype control (R&D Systems). In some experiments, leptin-stimulated RA-FLS were handled in the presence or absence of ETO. And in other experiments, recombinant human leptin protein (leptin, R&D Systems)-stimulated RA-FLS was treated with or without AMPK inhibitor, compound C (CC, Cell Signaling Technology).

### Isolation, culture and identification of FLS

In the experiment, we not only used immortalized cell lines, but also applied primary cells isolated from synovial tissues (~1 mm^3^) of RA patients. Primary RA-FLS were isolated as described previously [16]. Morphology of FLS was confirmed under the light microscope (Olympus Corporation, Japan) and further characterized by flow-cytometry (Agilent, USA) with following fluorescein-labeled antibodies: CD90-APC, CD73-PE, CD14-PE and CD34-APC (eBioscience, USA). Isotype-matched control antibodies were used as methodology controls. Stained cells were then examined using flow-cytometry and data analyzed by NovoExpress software (Agilent, USA). All experiments were performed with FLS after passage four (>95% FLS purity).

### RNA isolation and real-time PCR

Total mRNA was extracted from RA-FLS or HSF utilizing RNAiso Plus (TaKaRa Biotechnology Co., Ltd., Dalian, China) according to the manufacturer’s protocol and converted to cDNA using 5×All-In-One RT masterMix (Applied Biological Materials Inc, Canada). All qPCR reactions were carried out in triplicate using the CFX96 Real-Time PCR Detection System (Bio-Rad, USA). Quantitative measurement detection system of specific gene expression included 1 µL cDNA, 5 µL 2×SYBR Green mix (Accurate Biology, China) and 1 µL primer mixture. Primer sequences (Invitrogen, USA) used in this study were listed in Table S2. The reactions were incubated in a 96-well plate at 95 °C for 30 s, followed by 40 cycles of 95 °C for 5 s and 60 °C for 30 s. The cycle thresholds (CT) of the target gene were normalized to the CT value of the internal reference β-actin gene using the standard 2^−△△CT^ method.

### Measurements of cellular ATP

Cellular ATP was measured by an ATP Content Assay Kit (Solarbio, Beijing, China) following manufacturer’s instructions. Cells were plated at ~10^6^ cells in 75 cm^2^ culture bottles before the experiment. After treatment, cells were ultrasonically crushed and centrifuged at 10,000 g at 4 °C for 3 min. Collect the supernatant and detect ATP level. The content of ATP was determined by colorimetric method at 340 nm by Microplate Reader (Thermo Fisher Scientific, Inc. USA). Total ATP levels were reported as μmol/10^6^ cells.

### Western blot

The cells were washed twice with cold phosphate-buffered saline (PBS) and then dissolved in RIPA lysis buffer (Beyotime Biotechnology, China). After measurement of protein concentrations with BCA protein Assay kit (Nanjing KeyGen Biotech, China), equal amounts of protein (~20 μg) were separated by 12% sodium dodecyl sulfate-polyacrylamide gel electrophoresis (SDS-PAGE) and electrophoretically transferred to nitrocellulose filter (NC) membranes. Membranes were incubated with primary antibodies including CPT-1A (cat. no. 12252 S, diluted 1:1500), LKB1 (cat. no. 3050, diluted 1:1000), phospho-AMPK (cat. no. 2535 S, diluted 1:1000), AMPK(cat. no. 5831 S, diluted 1:1000), β-actin (cat. no. 4970 S, diluted 1:1000) and GAPDH (cat. no. 2188 S, diluted 1:1000) from Cell Signaling Techology, together with antibodies against CAMKK (cat. no. WL03453, Wanleibio, China, diluted 1:2000) and leptin receptor (cat. no. WL0162a, Wanleibio, China, diluted 1:1000) respectively at 4 °C overnight. Washed unbound antibody away with tris-buffered saline tween (TBST) solution and incubated with a fluorescent secondary antibody (cat. no. AS014, Abclonal, China, diluted 1:15,000) for 2 h at room temperature. Odyssey CLx Infrared Scanner (Odyssey CLx, USA) was utilized to detect the results, and ImageJ software was served for calculating relative protein expression.

### Immunofluorescence staining assay

Expression of leptin receptor was detected by immunofluorescent test upon RA serum stimulation. RA-FLS were planted on cover slips rinsed with PBS at the density of 2 × 10^4^ cells in 6-well plates. Fix the cells with freshly made 4% formaldehyde for 20 min after they have grown by adherence and wash gently with PBS. Incubate with anti-human leptin receptor antibody (cat. no. 130-125-241, Miltenyi Biotec, Germany, 1:100 diluted) at room temperature for 30 min. The nuclei was subsequently counterstained with Hoechst 33258 (Beyotime Biotechnology, China) for 5 min and washed with PBS. And subsequently, images were acquired under a fluorescence microscope (Olympus BX53, Japan) at an excitation wavelength of 480 nm.

### Carboxyfluorescein succinimidyl ester (CFSE) proliferation assay

FLS proliferation stimulated by RA serum or leptin (100 ng/mL) was assessed by CFSE dye (eBioscience, USA). CFSE is a fluorescent probe used to label live cells that were taken to daughter cells in the same way during division. Single-cell suspension of RA-FLS was tagged with CFSE (10 μM) for 10 min in the dark at 37 °C. Wash cells with culture media to remove unincorporated CFSE. Flow cytometry was used to analyze RA-FLS division tracking.

### Cell migration assay

The effect of leptin on vertical migration of primary RA-FLS was detected through transwell assays. Migration assay was performed using transwell chambers with 8 μm pores (BIOFIL, China) in 12-well plates. 2 × 10^4^ cells were re-suspended in serum-free medium and seeded in the upper chamber, while the lower chamber was filled with complete medium. After 24 h by treatment with leptin, the cells in the upper chamber were carefully removed with a cotton swab, and the migrated cells on the lower side of the membranes were fixed with methyl alcohol for 10 min and stained with 0.1% crystal violet. Migrated cells were counted from five random fields (×100). Images were taken using a microscope (Olympus BX53, Japan).

### HUVEC tube formation assay

The tube formation assay was performed as described previously [42]. A prechilled 96-well plate was filled with 50 μL/well of cold matrigel (10 mg/mL, Becton, Dickinson and Company, USA) and polymerized at 37 °C for 1 h. Serum-starved HUVEC were re-suspended in a 1:1 mixture of RA-FLS supernatant and then planted at a density of 2 × 10^4^ cells/well on the top of matrigel at 37 °C. Tube formation was examined every hour after cell seeding. Representative images were taken at 6 h with an inverted microscope (×100, NiKon, Japan) and the lengthened tubes were analyzed with ImageJ software.

### Cell adhesion assay

In order to identify the effects of FLS handled in different ways on monocyte adhesion, we establish the co-culutre system of FLS and THP-1. RA-FLS (4 × 10^4^), inoculated in 20 mm thick glass petri dish, were pre-stimulated by serum from RA patient, healthy control and leptin (100 ng/mL) separately for 24 h.

Then THP-1 cells were collected and labeled for 1 h with Invitrogen’s Intracellular pH Calibration Buffer Kit (Carlsbad, CA, USA). After that, 2 × 10^5^ THP-1 cells were added to the RA-FLS culture system after being washed twice and incubated for 6 h at 37 °C. The fluorescence was measured using a fluorescence microscope after nonadherent cells were rinsed away.

### Osteoclast formation assay

We examined the ability of FLS inducing osteoclast formation under leptin stimulation. THP-1 was cultured at a density of 5 × 10^5^ cells/well in 12-well plates. The supernatant of RA-FLS pre-treated by leptin (100 ng/mL) with or without ETO (dissolved in PBS with the working concentration of 100 μM) for 24 h was then applied to stimulate THP-1, 50 ng/mL receptor activator for nuclear factor-κB ligand (RANKL) (PeproTech Inc, USA) and 50 ng/mL macrophage colony stimulating factor (M-CSF) (PeproTech Inc, USA) were also added to the system for 15 days. The intercellular space was blurred under microscope, and polynuclear giant cells were united with each other.

Tartrate-resistant acid phosphatase (TRAP) staining was performed with a TRAP staining kit (Solarbio, China) according to the manufacturer’s instructions. Remove the culture medium and add 200 μL fixative solution for 3 min. The cells were then dyed with TRAP incubation solutions at 37 °C for 1 h. Finally, cells dyed red can be directly observed and calculated under a light microscope after cleaning with PBS (×100, NiKon, Japan).

### Protein–protein interaction network

In order to identify the specific pathway of leptin regulating fatty acid oxidation in RA-FLS cells, we investigated the interactions of leptin, its receptors and the main metabolic pathway-molecules, including mTOR, PI3K, AKT, AMPK, IRS, PPAR, APOA1, FDFT1, LKB1 and CAMKK with protein–protein interaction network system. All of the human protein–protein interactions were downloaded from STRING online analysis system. Only median confidence level of protein interactions is >0.4 was selected for further investigation.

### Cell transfection

RA-FLS (2 × 10^5^ cells/well) were seeded in 6-well culture plates with DMEM medium containing 10% FBS. Small interfering RNA (siRNA) for LKB1 and non-targeting negative control (NC) from General Biosystems of China were transient transfected into RA-FLS with lipofectamine 2000 reagent (Invitrogen, USA) in serum-free medium at the final concentration of 100 nM according to the manufacturer’s protocol. Cells were harvested at 24 h (for gene detection) and 48 h (for protein detection) following transfection for further analysis. SiRNA sequences for LKB1 (si-1261) were described as forward-5'-GGGCCAAGCUCAUCGGCAATT-3', reverse-5'-UUGCCGAUGAGCUUGGCCCTT-3'.

### Statistical analysis

Prism software (version 5, GraphPad) was used for all statistical analyses. The experimental data were presented as mean ± SEM based on ≥3 replicates. For statistical evaluation, the comparison between two groups was analyzed by *t*-test with Graphpad Prism 5.0. Statistical differences among groups were tested by one-way analysis of variance (ANOVA). All experimental data were independent repeated at least three times. Difffferences were considered statistically signifificant when *P* < 0.05.

## Supplementary information

checklist

supplemental legends

supplemental table

supplemental figure 1

supplemental figure 2

Original Data File

## Data Availability

The experimental datasets generated and/or analyzed during the current study are available from the corresponding author upon reasonable request. No applicable resources were generated during the current study.

## References

[1] Liu H, Zhu Y, Gao Y, Qi D, Zhao L, Zhao L (2020). NR1D1 modulates synovial inflammation and bone destruction in rheumatoid arthritis. Cell Death Dis.

[2] Bartok B, Firestein GS (2010). Fibroblast‐like synoviocytes: key effector cells in rheumatoid arthritis. Immunol Rev.

[3] Aghakhani S, Zerrouk N, Niarakis A (2020). Metabolic reprogramming of fibroblasts as therapeutic target in rheumatoid arthritis and cancer: deciphering key mechanisms using computational systems biology approaches. Cancers (Basel).

[4] Noack M, Miossec P (2021). Importance of lymphocyte-stromal cell interactions in autoimmune and inflammatory rheumatic diseases. Nat Rev Rheumatol.

[5] Bottini N, Firestein GS (2013). Duality of fibroblast-like synoviocytes in RA: passive responders and imprinted aggressors. Nat Rev Rheumatol.

[6] Yoshitomi H (2019). Regulation of immune responses and chronic inflammation by fibroblast-like synoviocytes. Front Immunol.

[7] Müller-Ladner U, Gay RE, Gay S (2000). Activation of synoviocytes. Curr Opin Rheumatol.

[8] Ng CT, Biniecka M, Kennedy A, McCormick J, Fitzgerald O, Bresnihan B (2010). Synovial tissue hypoxia and inflammation in vivo. Ann Rheum Dis.

[9] Rodgers LC, Cole J, Rattigan KM, Barrett MP, Kurian N, McInnes IB (2020). The rheumatoid synovial environment alters fatty acid metabolism in human monocytes and enhances CCL20 secretion. Rheumatol (Oxf).

[10] Xiong J (2018). Fatty acid oxidation in cell fate determination. Trends Biochem Sci.

[11] Tang M, Dong X, Xiao L, Tan Z, Luo X, Yang L (2022). CPT1A-mediated fatty acid oxidation promotes cell proliferation via nucleoside metabolism in nasopharyngeal carcinoma. Cell Death Dis.

[12] Sanchez-Lopez E, Cheng A, Guma M (2019). Can metabolic pathways be therapeutic targets in rheumatoid arthritis?. J Clin Med.

[13] Chihara K, Hattori N, Ichikawa N, Matsuda T, Saito T (2020). Re-evaluation of serum leptin and adiponectin concentrations normalized by body fat mass in patients with rheumatoid arthritis. Sci Rep..

[14] Conde J, Scotece M, Gómez R, Gómez-Reino JJ, Lago F, Gualillo O (2010). At the crossroad between immunity and metabolism: focus on leptin. Expert Rev Clin Immunol.

[15] Arora H, Qureshi R, Khodamoradi K, Seetharam D, Parmar M, Van Booven DJ (2022). Leptin secreted from testicular microenvironment modulates hedgehog signaling to augment the endogenous function of Leydig cells. Cell Death Dis.

[16] Sun X, Wei J, Tang Y, Wang B, Zhang Y, Shi L (2017). Leptin-induced migration and angiogenesis in rheumatoid arthritis is mediated by reactive oxygen species. FEBS Open Bio.

[17] Kelesidis T, Kelesidis I, Chou S, Mantzoros CS (2010). Narrative review: the role of leptin in human physiology: Emerging clinical applications. Ann Intern Med.

[18] Bustamante MF, Garcia-Carbonell R, Whisenant KD, Guma M (2017). Fibroblast-like synoviocyte metabolism in the pathogenesis of rheumatoid arthritis. Arthritis Res Ther.

[19] Wang T, Fahrmann JF, Lee H, Li YJ, Tripathi SC, Yue C (2018). JAK/STAT3-regulated fatty acid β-oxidation is critical for breast cancer stem cell self-renewal and chemoresistance. Cell Metab.

[20] Wang M, Wei J, Li H, Ouyang X, Sun X, Tang Y (2018). Leptin upregulates peripheral CD4+CXCR5+ICOS+ T cells via increased IL-6 in rheumatoid arthritis patients. J Interferon Cytokine Res.

[21] Carracedo A, Cantley LC, Pandolfi PP (2013). Cancer metabolism: fatty acid oxidation in the limelight. Nat Rev Cancer.

[22] Qu Q, Zeng F, Liu X, Wang QJ, Deng F (2016). Fatty acid oxidation and carnitine palmitoyltransferase I: emerging therapeutic targets in cancer. Cell Death Dis.

[23] Fujikawa Y, Sabokbar A, Neale S, Athanasou NA (1996). Human osteoclast formation and bone resorption by monocytes and synovial macrophages in rheumatoid arthritis. Ann Rheum Dis.

[24] Auréal M, Machuca-Gayet I, Coury F (2020). Rheumatoid arthritis in the view of osteoimmunology. Biomolecules.

[25] Shaw RJ, Kosmatka M, Bardeesy N, Hurley RL, Witters LA, DePinho RA (2004). The tumor suppressor LKB1 kinase directly activates AMP-activated kinase and regulates apoptosis in response to energy stress. Proc Natl Acad Sci USA.

[26] Fogarty S, Ross FA, Vara Ciruelos D, Gray A, Gowans GJ, Hardie DG (2016). AMPK causes cell cycle arrest in LKB1-deficient cells via activation of CAMKK2. Mol Cancer Res.

[27] Du Y, Wang Q, Tian N, Lu M, Zhang XL, Dai SM. Knockdown of nrf2 exacerbates TNF-α-induced proliferation and invasion of rheumatoid arthritis fibroblast-like synoviocytes through activating JNK pathway. J Immunol Res. 2020;2020:6670464. 10.1155/2020/6670464 PMC7772017 33426091

[28] He L, Luan H, He J, Zhang M, Qin Q, Hu Y (2021). Shikonin attenuates rheumatoid arthritis by targeting SOCS1/JAK/STAT signaling pathway of fibroblast like synoviocytes. Chin Med.

[29] Lou L, Liu Y, Zhou J, Wei Y, Deng J, Dong B (2015). Chlorogenic acid and luteolin synergistically inhibit the proliferation of interleukin-1β-induced fibroblast-like synoviocytes through regulating the activation of NF-κB and JAK/STAT-signaling pathways. Immunopharmacol Immunotoxicol.

[30] Huang SC, Evert B, Ivanova Y, O’Sullivan D, Nascimento M, Smith AM (2014). Cell-intrinsic lysosomal lipolysis is essential for alternative activation of macrophages. Nat Immunol.

[31] Nomura M, Liu J, Rovira II, Gonzalez-Hurtado E, Lee J, Wolfgang MJ (2016). Fatty acid oxidation in macrophage polarization. Nat Immunol.

[32] Carlton ED, Demas GE, French SS (2012). Leptin, a neuroendocrine mediator of immune responses, inflammation, and sickness behaviors. Horm Behav.

[33] La Cava A, Matarese G (2004). The weight of leptin in immunity. Nat Rev Immunol.

[34] Tian Z, Sun R, Wei H, Gao B (2002). Impaired natural killer (NK) cell activity in leptin receptor deficient mice: leptin as a critical regulator in NK cell development and activation. Biochem Biophys Res Commun.

[35] Wang X, Qiao Y, Yang L, Song S, Han Y, Tian Y (2017). Leptin levels in patients with systemic lupus erythematosus inversely correlate with regulatory T cell frequency. Lupus.

[36] Jarecki J, Małecka-Massalska T, Polkowska I, Potoczniak B, Kosior-Jarecka E, Szerb I (2021). Level of adiponectin, leptin and selected matrix metalloproteinases in female overweight patients with primary gonarthrosis. J Clin Med.

[37] Taylan A, Akinci B, Toprak B, Birlik M, Arslan FD, Ekerbicer H (2021). Association of leptin levels and disease activity in patients with early rheumatoid arthritis. Arch Med Res.

[38] Chen Z, Xing T, Li J, Zhang L, Jiang Y, Gao F (2022). Oxidative stress induced by hydrogen peroxide promotes glycosis by activating CaMKK/LKB1/AMPK pathway in broiler breast muscle. Poult Sci.

[39] Carling D (2017). AMPK signalling in health and disease. Curr Opin Cell Biol.

[40] Subramanian A, Tamayo P, Mootha VK, Mukherjee S, Ebert BL, Gillette MA (2005). Gene set enrichment analysis: a knowledge-based approach for interpreting genome-wide expression profiles. Proc Natl Acad Sci USA.

[41] Arnett FC, Edworthy SM, Bloch DA, Mcshane DJ, Fries JF, Cooper NS (1988). The American Rheumatism Association 1987 revised criteria for the classification of rheumatoid arthritis. Arthritis Rheum.

[42] Cavallari G, Olivi E, Bianchi F, Neri F, Foroni L, Valente S (2012). Mesenchymal stem cells and islet cotransplantation in diabetic rats: improved islet graft revascularization and function by human adipose tissue-derived stem cells preconditioned with natural molecules. Cell Transpl.

