# Supplementary material for: Elevated fatty acid β-oxidation by leptin contributes to the proinflammatory characteristics of fibroblast-like synoviocytes from RA patients via LKB1-AMPK pathway
Source: Cell Death Dis. 2023 Feb 9;14(2):97. doi: 10.1038/s41419-023-05641-2 (PMC9911755; doi:10.1038/s41419-023-05641-2)
Supplement: Supplementary file 2 — supplemental legends [file 41419_2023_5641_MOESM2_ESM.docx]

**Supplementary Figure 1 (Fig. S1). Phenotype identification of RA-FLS.** RA-FLS were incubated with antibodies against CD14, CD34, CD73 and CD90 for 30 minutes then analyzed by flow cytometry. The figure showed that CD73 and CD90 markers were positive, whereas CD14, and CD34 markers were negative. Data are means ± SD of three independent experiments.

**Supplementary Figure 2 (Fig. S1). Silencing effect of LKB1 triggered by small interfering RNA in RA-FLS.** Gene (upper) and protein (lower) expression of LKB1 following transfection with different siRNAs by qRT-PCR and westernblot. Data are means ± SD of three independent experiments. siRNA, small interfering RNA; NC, untransfected cells.
