# Supplementary material for: Elevated fatty acid β-oxidation by leptin contributes to the proinflammatory characteristics of fibroblast-like synoviocytes from RA patients via LKB1-AMPK pathway
Source: Cell Death Dis. 2023 Feb 9;14(2):97. doi: 10.1038/s41419-023-05641-2 (PMC9911755; doi:10.1038/s41419-023-05641-2)
Supplement: Supplementary file 3 — supplemental table [file 41419_2023_5641_MOESM3_ESM.docx]

**Supplementary Table 1 (Table S1). Details of datasets used in this study**

| **CEO accession** |  | **Timing of gene expression profiling** | **Country** | **Sample number** | |  |
| --- | --- | --- | --- | --- | --- | --- |
|  |  |  |  | **RA** | **Normal** |  |
| GSE48780 | Expression date from Rheumatoid Arthritis  synovial tissue samples | At enrollment | USA | 83 | 0 | |
|  |  |  |  |  |  |  |
| GSE89408 | Total RNA sequencing was performed on samples isolated from joint synovial biopsies from subjects with and without Rheumatoid Arthritis | At enrollment | USA | 152 | 28 |  |

**Supplementary Table 2 (Table S2). Primer sequences used for cDNA amplification**

| **Gene** | **Forward** | **Reverse** |
| --- | --- | --- |
| β-actin | 5′-CATGTACGTTGCTATCCAGGC-3′ | 5′-CTCCTTAATGTCACGCACGAT-3′ |
| CPT-1A | 5′-TCCAGTTGGCTTATCGTGGTG-3′ | 5′-TCCAGAGTCCGATTGATTTTTGC-3′ |
| CPT-1B | 5′-CCTGCTACATGGCAACTGCTA-3′ | 5′-AGAGGTGCCCAATGATGGGA |
| CPT-1C | 5′- GGGCCGCTTTCTTTGTGTC-3′ | 5′-AGAAGACGATTAGGGTGAAGGAT-3′ |
| CPT-2 | 5′-CTGGAGCCAGAAGTGTTCCAC-3′ | 5′-AGGCACAAAGCGTATGAGTCT-3′ |
| ACAD11 | 5′-TTGGATTCCCCGTTCCCAAG-3′ | 5′-AAATCACGGAAGATTCGACCC-3′ |
| HAD-HA | 5′-ATATGCCGCAATTTTACAGGGT-3′ | 5′-ACCTGCAATAAAGCAGCCTGG-3′ |
| HAD-HB | 5′-TACGGGTTTGTTGCATCGGAC-3′ | 5′-GCCACATTGCTTGTTTTCACTT-3′ |
| Leptin Receptor | 5′-GCATGCAGAATCAGTGATATTTGG-3′ | 5′-CAAGCTGTATCGACACTGATTTCTTC-3′ |
| TNF-α | 5′-CGAGTGACAAGCCTGTAGC-3′ | 5′-GGTGTGGGTGAGGAGCACAT-3′ |
| IL-1β | 5′-GCTGAGGAAGATGCTGGTTC-3′ | 5′-GTGATCGTACAGGTGCATCG-3′ |
| VEGF | 5′-GAGGGCAGAATCATCACGAAGTGG-3′ | 5′-ATCGCATCAGGGGCACACAGGAT-3′ |
| IL-6  ICAM-1  VCAM-1  CCL2  CX3CL1  RANKL  OPG  GAPDH | 5′-CCTTCGGTCCAGTTGCCTTCTC-3′  5′-GCAAGAAGATAGCCAACCA-3′  5′-ACCACATCTACGCTGACAATGAATCC-3′  5′-CAGCCAGATGCAATCAATGCC-3′  5′-CACCTTCTGCCATCTGACTGT-3′  5′-GCGTCGCCCTGTTCTTCTAT -3′  5′-GCTTGAAACATAGGAGCTG -3′  5′-CGAGATCCCTCCAAAATCAA -3′ | 5′-CCAGTGCCTCTTTGCTGCTTTC-3′  5′-TGCCAGTTCCACCCGTTC-3′  5′-AACACTTGACTGTGATCGGCTTCC-3′  5′-TGGAATCCTGAACCCACTTCT-3′  5′-GCATGATGCCTGGTTCTGTTG-3′  5′-TGCAGTGAGTGCCATCTTCTG -3′  5′-GTTTACTTTGGTGCCAGG -3′  5′-TTCACACCCATGACGAACAT -3′ |

**Supplementary Table 3 (Table S3). Protein interactions among leptin, leptin receptor and major metabolic pathway-proteins**

| **Node1** | **Node2** | **Phylogenetic**  **cooccurrence** | **Homology** | **Coexpression** | **Experimentally**  **determined**  **interaction** | **Database**  **annotated** | **Automated**  **textmining** | **Combined**  **score** |
| --- | --- | --- | --- | --- | --- | --- | --- | --- |
| AKT1 | PRKAB1 | 0 | 0 | 0 | 0.114 | 0 | 0.426 | 0.47 |
| AKT1 | APOA1 | 0 | 0 | 0 | 0 | 0 | 0.448 | 0.448 |
| AKT1 | PIK3CA | 0 | 0 | 0.064 | 0.88 | 0.9 | 0.903 | 0.998 |
| AKT1 | LEP | 0 | 0 | 0 | 0 | 0 | 0.739 | 0.739 |
| AKT1 | STK11 | 0.325 | 0.631 | 0.104 | 0.298 | 0 | 0.807 | 0.588 |
| AKT1 | LEPR | 0 | 0 | 0 | 0.056 | 0 | 0.571 | 0.578 |
| AKT1 | MTOR | 0 | 0 | 0.081 | 0.904 | 0.9 | 0.991 | 0.999 |
| AKT1 | PPARA | 0 | 0 | 0 | 0.077 | 0 | 0.686 | 0.698 |
| APOA1 | STK11 | 0 | 0 | 0 | 0 | 0 | 0.432 | 0.432 |
| APOA1 | LEP | 0 | 0 | 0 | 0 | 0 | 0.609 | 0.609 |
| APOA1 | PPARA | 0 | 0 | 0.064 | 0 | 0.9 | 0.704 | 0.969 |
| CAMKK2 | PRKAB1 | 0 | 0 | 0.064 | 0.244 | 0.9 | 0.696 | 0.975 |
| CAMKK2 | MTOR | 0 | 0 | 0.062 | 0 | 0 | 0.549 | 0.559 |
| CAMKK2 | STK11 | 0 | 0.705 | 0.064 | 0.256 | 0.8 | 0.852 | 0.886 |
| FDFT1 | PPARA | 0 | 0 | 0 | 0 | 0.9 | 0.323 | 0.929 |
| LEP | PRKAB1 | 0 | 0 | 0 | 0 | 0.9 | 0.194 | 0.915 |
| LEP | STK11 | 0 | 0 | 0 | 0 | 0 | 0.501 | 0.501 |
| LEP | MTOR | 0 | 0 | 0 | 0 | 0 | 0.684 | 0.685 |
| LEP | PPARA | 0 | 0 | 0 | 0 | 0 | 0.772 | 0.772 |
| LEP | LEPR | 0 | 0 | 0 | 0.735 | 0.9 | 0.992 | 0.999 |
| LEPR | PRKAB1 | 0 | 0 | 0 | 0 | 0.9 | 0.102 | 0.906 |
| LEPR | MTOR | 0 | 0 | 0 | 0 | 0 | 0.429 | 0.429 |
| LEPR | PPARA | 0 | 0 | 0 | 0 | 0 | 0.571 | 0.571 |
| MTOR | PRKAB1 | 0 | 0 | 0.067 | 0.122 | 0.9 | 0.494 | 0.953 |
| MTOR | PIK3CA | 0 | 0.541 | 0.062 | 0.347 | 0.9 | 0.782 | 0.957 |
| MTOR | STK11 | 0 | 0 | 0.062 | 0.175 | 0 | 0.762 | 0.799 |
| MTOR | PPARA | 0 | 0 | 0 | 0.061 | 0 | 0.559 | 0.568 |
| PIK3CA | STK11 | 0 | 0 | 0.083 | 0.21 | 0 | 0.716 | 0.776 |
| PPARA | STK11 | 0 | 0 | 0 | 0.058 | 0 | 0.507 | 0.515 |
| PRKAB1 | STK11 | 0 | 0 | 0.062 | 0.343 | 0.9 | 0.661 | 0.976 |
