# Supplementary figures and images for: Elevated fatty acid β-oxidation by leptin contributes to the proinflammatory characteristics of fibroblast-like synoviocytes from RA patients via LKB1-AMPK pathway
Source: Cell Death Dis. 2023 Feb 9;14(2):97. doi: 10.1038/s41419-023-05641-2 (PMC9911755; doi:10.1038/s41419-023-05641-2)

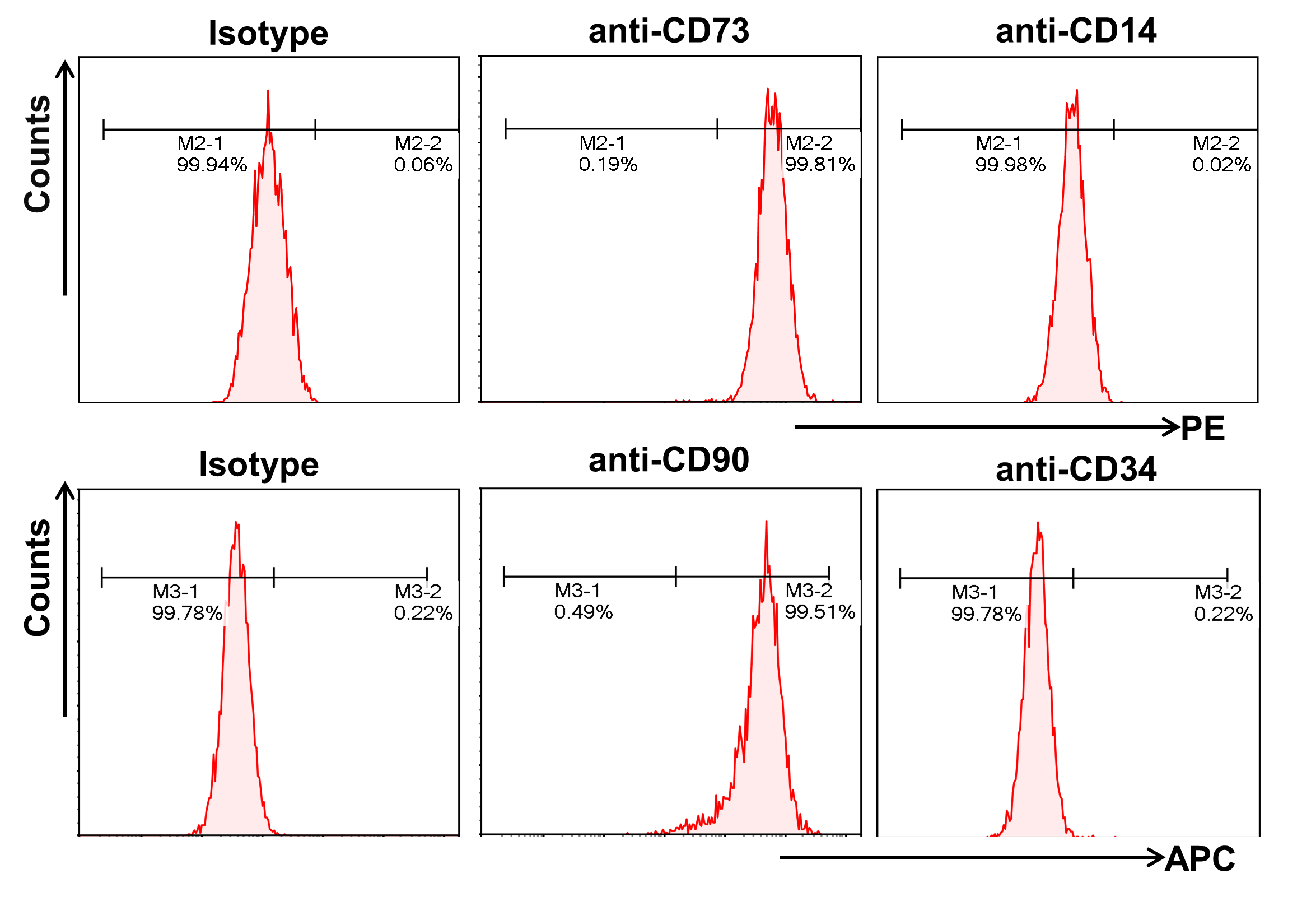

Supplement: Supplementary file 4 — supplemental figure 1 [file 41419_2023_5641_MOESM4_ESM.png]

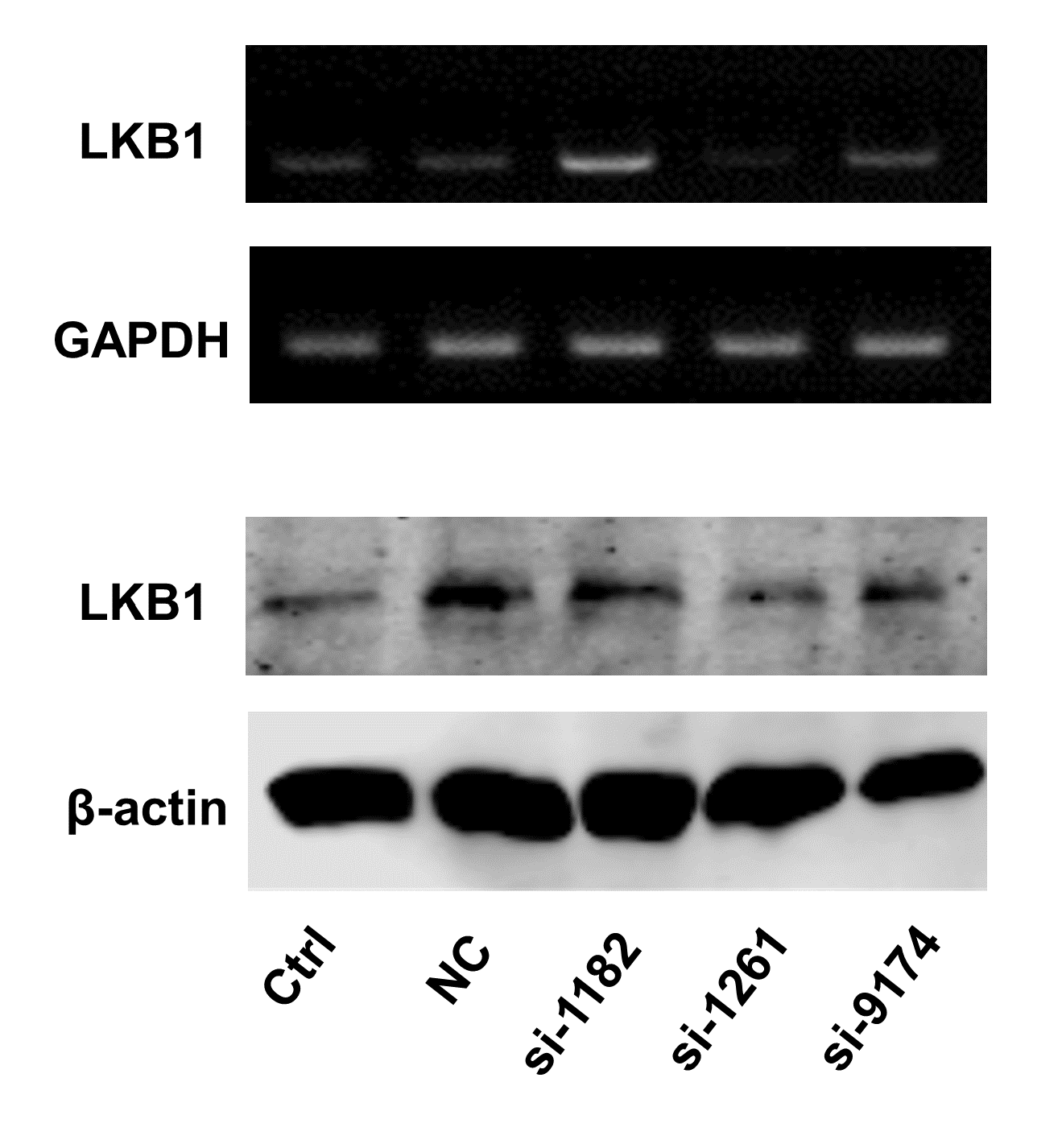

Supplement: Supplementary file 5 — supplemental figure 2 [file 41419_2023_5641_MOESM5_ESM.png]
