# Supplementary material for: Elevated fatty acid β-oxidation by leptin contributes to the proinflammatory characteristics of fibroblast-like synoviocytes from RA patients via LKB1-AMPK pathway
Source: Cell Death Dis. 2023 Feb 9;14(2):97. doi: 10.1038/s41419-023-05641-2 (PMC9911755; doi:10.1038/s41419-023-05641-2)
Supplement: Supplementary file 6 — Original Data File [file 41419_2023_5641_MOESM6_ESM.pptx]

## Slide 1
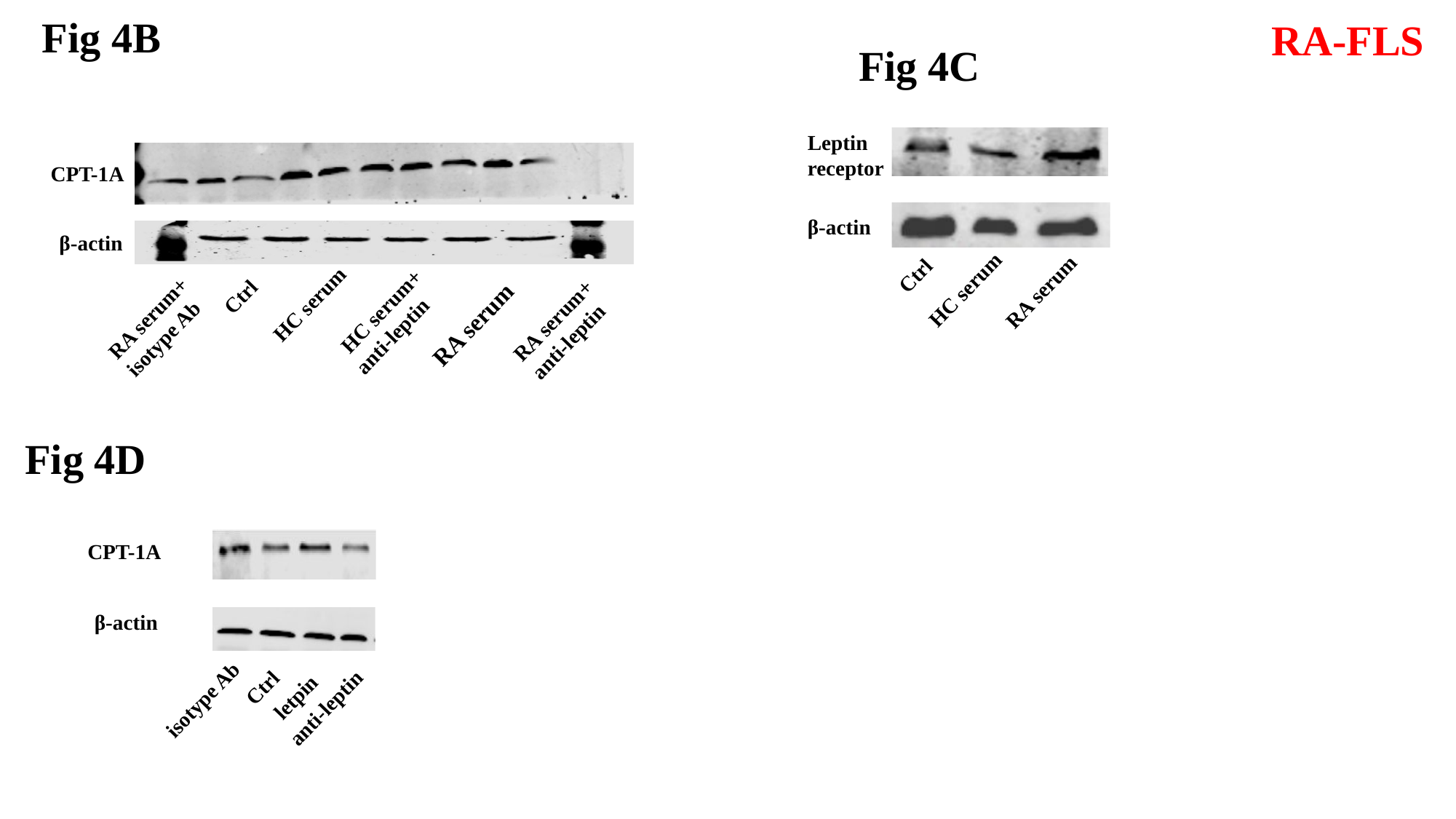

Fig 4B
RA-FLS
Fig 4C
Leptin receptor
CPT-1A
β-actin
β-actin
 Ctrl
RA serum
 HC serum
 Ctrl
 HC serum
 HC serum+
anti-leptin
RA serum+
isotype Ab
RA serum+
anti-leptin
RA serum
Fig 4D
CPT-1A
β-actin
 Ctrl
 letpin
isotype Ab
anti-leptin

## Slide 2
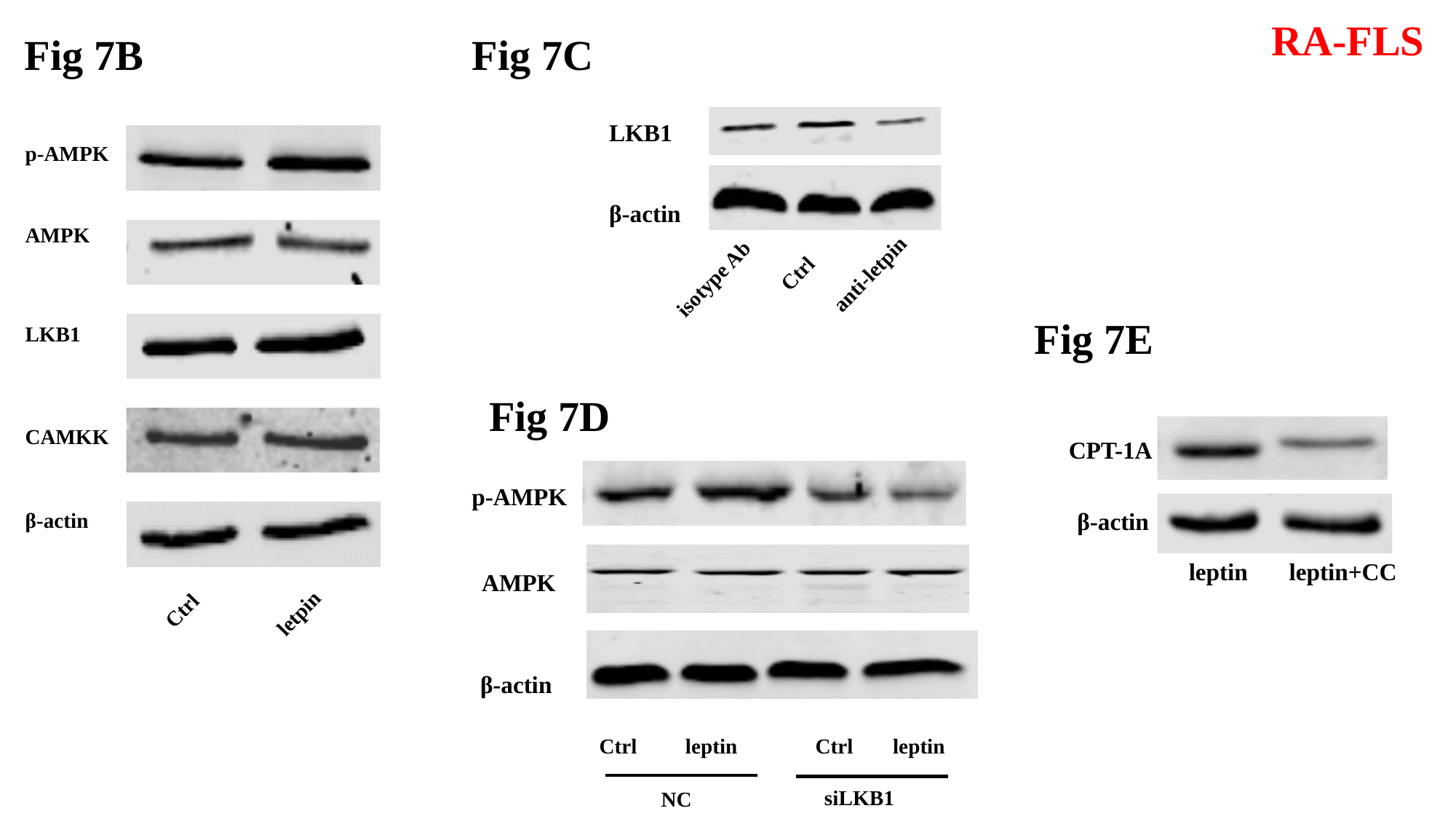

RA-FLS
Fig 7C
Fig 7B
LKB1
p-AMPK
β-actin
AMPK
 Ctrl
 anti-letpin
 isotype Ab
Fig 7E
LKB1
Fig 7D
CAMKK
CPT-1A
p-AMPK
β-actin
β-actin
leptin
leptin+CC
AMPK
 Ctrl
 letpin
β-actin
Ctrl
leptin
Ctrl
leptin
siLKB1
NC

## Slide 3
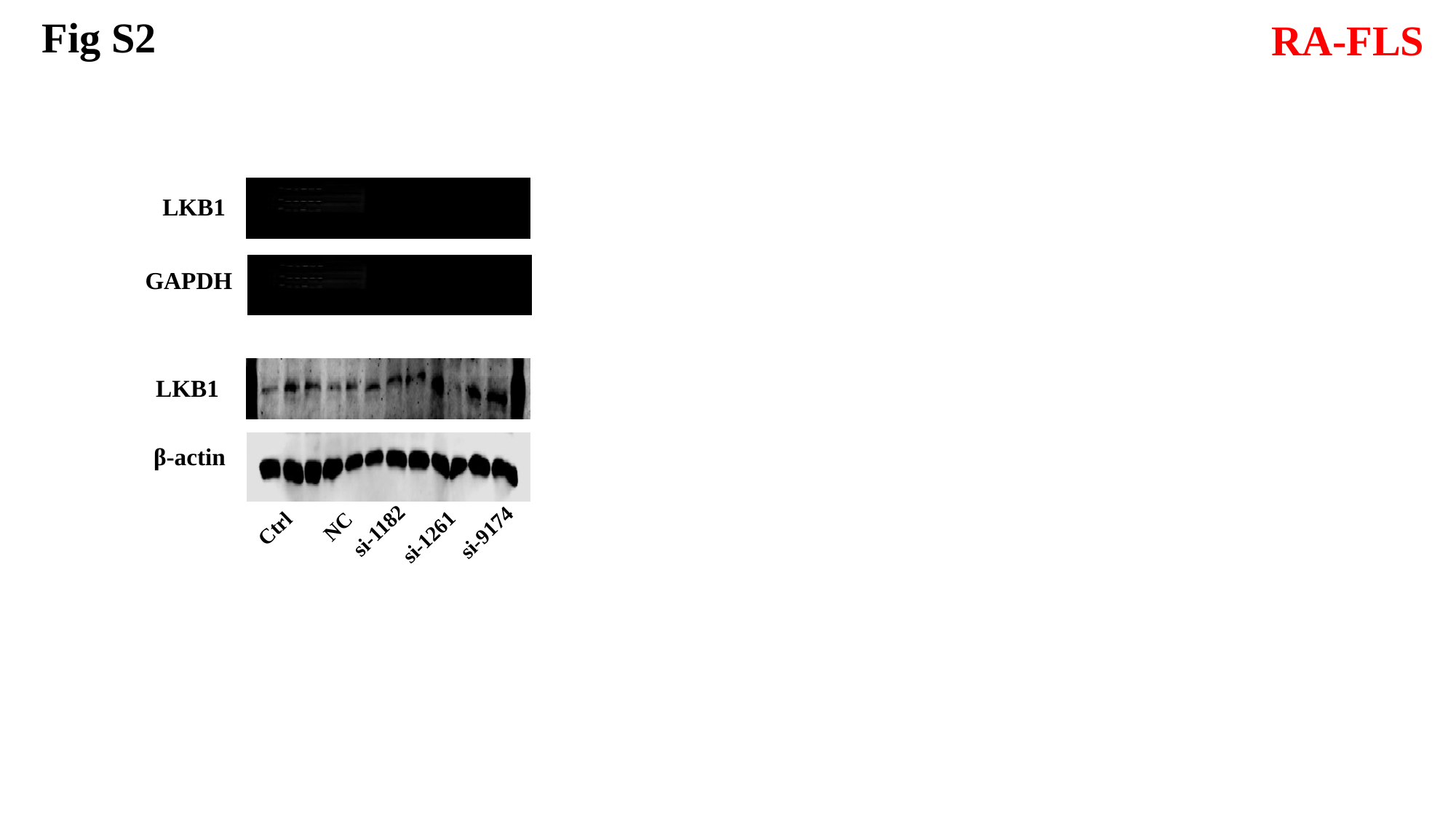

Fig S2
RA-FLS
LKB1
GAPDH
LKB1
β-actin
NC
Ctrl
si-1182
si-9174
si-1261
